# Sinicization and validation of occupational burnout scale for nurses in operating room

**DOI:** 10.3389/fpubh.2025.1559204

**Published:** 2025-03-19

**Authors:** Yufang Li, Qiaoyan Liu, Yun Jiang, Deyun Cheng, Wei Yin

**Affiliations:** ^1^Department of Nursing, Affiliated Hospital of Jiangsu University, Zhenjiang, China; ^2^Nursing Department, Anqing Municipal Hospital, Anqing, Anhui, China

**Keywords:** operating room nurse, job burnout, reliability, validity, scale

## Abstract

**Objective:**

This study aims to evaluate a burnout scale specifically developed for operating room nurses.

**Methods:**

The Brislin translation model was utilized to translate and back-translate the scale. Following cultural adaptation and a preliminary investigation, the Chinese version of the Operating Room Nurse Burnout Scale was finalized. A convenience sampling method was employed to select 445 operating room nurses in Anhui Province as research participants to evaluate the scale's reliability and validity.

**Results:**

The Chinese version of the Operating Room Nurse Burnout Scale consists of 33 items, categorized into four dimensions: personal factors, occupational nature factors, interpersonal relationship factors, and organizational factors. The item-level content validity index (I-CVI) was 0.849, and the scale-level content validity index (S-CVI/Ave) indicated good content validity. Exploratory factor analysis yielded a Kaiser-Meyer-Olkin (KMO) value of 0.968, while Bartlett's test of sphericity demonstrated a chi-square value of 11,288 (*p* < 0.01). Four common factors were extracted, accounting for a cumulative variance contribution of 65.9%. The overall Cronbach's α coefficient was 0.968, the split-half Cronbach's α coefficient was 0.925, and the test-retest Cronbach's α coefficient was 0.974.

**Conclusion:**

The Chinese version of the Nurse Burnout Scale demonstrates robust reliability and validity, making it suitable for assessing burnout levels among operating room nurses.

## 1 Introduction

Burnout was first introduced by American scholar Freudenberger (1) in 1974 as a state of physical and mental exhaustion caused by external factors. In 1981, Maslach and Jackson (2) described burnout as an individual's response to chronic work stress, comprising three components: emotional exhaustion, depersonalization, and low personal fulfillment—a psychological syndrome primarily resulting from chronic, long-term job stress (3). The high-intensity, high-risk, and high-stress nature of nursing makes this profession particularly susceptible to burnout. One study indicated that the prevalence of burnout among nurses worldwide is ~11.23% (4), while moderate and severe burnout rates in China are 25 and 12.5%, respectively (5). Burnout can lead to various physical and mental health issues, including insomnia, anxiety, depression, fatigue, and even suicidal tendencies. Additionally, it is closely associated with patient safety, quality of care, patient satisfaction, and turnover rates (6–9).

The operating room plays a critical role in providing surgical treatment and emergency care for patients, characterized by its specialization, complexity, and fast-paced environment. Operating room nurses are tasked with numerous responsibilities, including resuscitation, collaboration, and preparation for various intricate surgical procedures. Additionally, they are often involved in teaching and training activities, which can further increase their susceptibility to burnout. Recent findings (32) indicate that the incidence of severe emotional exhaustion, severe depersonalization, and significant feelings of low personal accomplishment among operating room nurses is concerning, with rates of 33.6, 24.8, and 47.3%, respectively. Given these statistics, it is crucial to prioritize the mental health of nurses working in the operating room and implement strategies to reduce burnout among this vital nursing staff.

Currently, the most commonly used assessment tool worldwide is the Maslach Burnout Scale (10), which was developed by Maslach and Jackson in 1981. This scale includes three versions, with the MBI-GS being the most widely utilized in the nursing field. The MBI-GS was adapted for Chinese use by scholars such as Chaoping (11) in 2003. After this adaptation, the Cronbach's α coefficients for the three dimensions of the scale exceeded 0.80, indicating good reliability and validity. However, the scale employs a 7-point Likert scale, which may be overly complex in describing frequency and has a low degree of differentiation, potentially preventing subjects from accurately selecting options that reflect their situations, thereby biasing the results. Additionally, the absence of reverse-scored items limits the validity of the test and may introduce errors in the findings (12). Other generalized scales, such as the Spanish Burnout Scale and the Copenhagen Burnout Inventory (CBI), have yet to demonstrate sensitivity and specificity in special populations. The Nurse Burnout Scale (NBS) was adapted for Chinese use by Tang et al. (13) in 2007; however, it has too many items with some redundancy, and no researchers in China have applied it specifically to operating room nurses (14). As a specialized department within hospitals, the factors influencing burnout among operating room nurses are unique, yet there is currently no specific measurement tool for assessing burnout in this population.

We aimed to examine the content validity, structural validity, exploratory factor analysis (EFA), internal consistency, split-half reliability, test-retest reliability, and the performance of the Chinese version of the Operating Room Nurse Burnout Scale. The study was conducted with a sample of operating room nurses from a tertiary care hospital in Anhui Province. The primary objective of this research is to develop an effective tool for assessing burnout among operating room nurses.

Teymoori et al. (15) developed the English version of the Operating Room Nurse Burnout Scale in 2024, which is user-friendly and has demonstrated good reliability and validity. This scale can be utilized to measure the degree of burnout among operating room nurses and to investigate the factors influencing burnout within this population. In this study, after obtaining consent from the authors of the original scale, the English version of the Operating Room Nurse Burnout Scale was adapted for use in China and tested for reliability. The aim is to provide a basis for nursing staff in China to select an appropriate burnout measurement tool for operating room nurses.

The purpose of this study was to translate the English version of the Operating Room Nurse Burnout Scale for cross-cultural adaptation and to provide an objective measurement of burnout among operating room nurses in the workplace. This research aims to raise awareness among nursing administrators about the psychological wellbeing of operating room nurses. Additionally, it serves as a reference for future preventive interventions aimed at enhancing the mental health of OR nurses.

## 2 Research methods

### 2.1 Sample

Nine experts were invited to evaluate the content validity of the Operating Room Nurse Burnout Scale. In this study, the convenience sampling method was employed to select operating room nurses working in a tertiary hospital in Anhui Province between July and August 2024. The inclusion and exclusion criteria were consistent with those used in the pre-survey. Based on the principle that the sample size should be 5–10 times the number of scale items and anticipating a 20% attrition rate, the estimated sample size required was at least 204 participants. This estimate takes into account that exploratory factor analysis typically requires more than 150 cases, and the Chinese version of the Operating Room Nurse Burnout Scale consists of 34 items (16). The study was approved by the Medical Ethics Committee under the number Medical Ethics Review (2024) No. 128. The demographic data of 445 nurses are shown in Table 1.

**Table 1:** General information on the respondents (*n* = 445).

**Characteristic**		**Nurses, *n* (%)**
Gender	Male	91 (20.4)
	Female	354 (79.6)
Age (years)	20–30	163 (36.6)
	31–40	235 (52.8)
	41–50	43 (9.7)
	>50	4 (0.9)
Marital status	Married	323 (72.6)
	Unmarried	114 (25.6)
	Divorced	8 (1.8)
Educational attainment	Specialized	63 (14.2)
	Undergraduate	374 (84.0)
	Master's degree	8 (1.8)
Job title	Physiotherapists	56 (12.6)
	Nurse practitioner	180 (40.4)
	Nurse practitioner in charge	189 (42.5)
	Associate nurse practitioner	19 (4.3)
	Chief nurse	1 (0.2)
Years of experience in the operating room (years)	≤ 5 years	130 (29.2)
	6–10 years	137 (30.8)
	11–20 years	153 (34.4)
	≥20 years	25 (5.6)
Is an operating room nurse specialist	Yes	70 (15.7)
	Negate	375 (84.3)
Duties	None	432 (97.1)
	Deputy nurse manager	6 (1.3)
	Nurse manager	6 (1.3)
	Section nurse manager	1 (0.2)
Gross household income	< 100,000	124 (27.9)
	100–200 thousand	219 (49.2)
	200,000–500,000	92 (20.7)
	>500,000	10 (2.2)
Whether or not they have received training related to burnout	Yes	62 (13.9)
	Negate	383 (86.1)

### 2.2 Procedure

#### 2.2.1 Translation of the scale

After obtaining authorization from the original authors, the original scale was adapted for Chinese use following the Brislin translation model (17). The original scale is in the Appendix 1.

##### 2.2.1.1 Direct translation

Two individuals were invited to independently translate the original scale into Chinese: one with a master's degree in nursing who had passed the sixth level of English, and one MD specializing in general surgery who had also passed the sixth level of English. This process resulted in two Chinese versions, referred to as Questionnaire 1 and Questionnaire 2. The researcher then integrated these two questionnaires and compared the Chinese versions with the original scale alongside the two translators, ultimately refining the translation to create the Chinese version, Questionnaire A, by addressing any ambiguities through discussion.

##### 2.2.1.2 Back-translation

Two translators proficient in both English and Chinese were invited to back-translate Questionnaire A. One translator held a PhD in English, while the other had a PhD in nursing with experience studying abroad. Neither expert had seen the original scale prior to this process. After completing the back-translation, the researcher synthesized the two English versions and facilitated a discussion between the back-translators to ensure consensus on the final back-translated version of Questionnaire A.

##### 2.2.1.3 Review by the original authors

The Chinese version of Questionnaire A and the back-translated version were sent to the original authors for review via email. Following their consultation and agreement, the final Chinese version, Questionnaire B, was established.

#### 2.2.2 Cultural debugging

Nine experts were invited for cultural validation, with the following inclusion criteria: a bachelor's degree or higher, a supervisory nurse position or above, and at least 10 years of experience in operating room specialties. The final expert panel consisted of two professors from the School of Nursing, six clinical nursing experts, and one nursing research expert. Among the panel members, one held a doctorate in nursing, six had master's degrees in nursing, and two had bachelor's degrees. Additionally, two experts held senior titles, while three held associate senior titles.

The Delphi method was employed for the evaluation of the Chinese version of Questionnaire B. Using a Likert scale, experts assessed the questionnaire items across various dimensions based on their theoretical knowledge and practical experience. They evaluated the importance of each item, the relevance of the content, the clarity of language expression, and the cultural compatibility of the items with the Chinese context. After thorough discussions and consultations, the final version of the Chinese questionnaire, referred to as Questionnaire B1, was established to ensure its validity and applicability.

#### 2.2.3 Pre-survey

In this study, 30 operating room nurses were selected for a pre-survey conducted in July 2024 at a tertiary hospital using a convenience sampling method. The inclusion criteria were nurses with more than 1 year of experience in operating room nursing, while the exclusion criteria included trainees and nurses who were absent from work due to illness or maternity leave. All participants voluntarily took part in this study based on informed consent.

### 2.3 Measures

#### 2.3.1 Survey instruments

##### 2.3.1.1 General information questionnaire

This questionnaire gathered demographic information from the participants, including age, gender, years of experience in operating room nursing, and whether they were nurses in a specialized operating room nursing department.

##### 2.3.1.2 Chinese version of the burnout scale for operating room nurses

This scale was used to assess the level of burnout among the operating room nurses.

#### 2.3.2 Introduction to the Operating Room Nurse Burnout Scale

The English version of the Burnout Scale for Operating Room Nurses consists of five dimensions: organizational factors, personal factors, interpersonal factors, occupational factors, and management factors, totaling 34 items. Each item is scored on a 5-point Likert scale, categorized into five responses: never, seldom, sometimes, often, and always, with corresponding scores ranging from 0 to 4. A higher score indicates a more severe degree of burnout. The Cronbach's alpha coefficient for the scale was found to be 0.937, and the test-retest reliability was 0.946, indicating excellent reliability.

### 2.4 Data collection and analysis

#### 2.4.1 Data collection method

Questionnaire Star was utilized for online data collection, with all questions designated as mandatory to ensure the completeness of responses. Each participant could submit the questionnaire only once to prevent duplicate entries. The researcher provided uniform instructions to guide the participants in completing the scale. Thirty randomly selected operating room nurses completed the scale again 2 weeks later to assess the retest validity of the instrument. In total, 506 questionnaires were collected for this study, of which 61 were deemed invalid (due to regular answers or short completion times), resulting in a valid questionnaire return rate of 87.94%.

#### 2.4.2 Statistical methods

Data were statistically analyzed using SPSS version 26.0 and AMOS version 24.0 software. Categorical data were described using frequencies and percentages, while continuous data were expressed as mean ± standard deviation.

#### 2.4.3 Item analysis

##### 2.4.3.1 Decision value method

The total scores of the Chinese version of the Operating Room Nurse Burnout Scale were sorted from low to high. The lowest 27% of scores were designated as the low group, while the highest 27% constituted the high group. An independent samples *t*-test was conducted on the scores of each item between the two groups. An item could be deleted if the difference between the groups was not significant (*P* > 0.05) or if the critical ratio value (CR) was < 3.0 (18).

##### 2.4.3.2 Correlation coefficient method

The correlation between the scores of each item on the scale and the total score of the scale, as well as the total score of each dimension, was analyzed. Items were retained if the correlation coefficient (r) was greater than or equal to 0.4 (*P* < 0.05); otherwise, they were deleted (19).

#### 2.4.4 Validity test

##### 2.4.4.1 Content validity

Nine experts were invited to evaluate the relevance of each item in the Chinese version of the Operating Room Nurses Burnout Scale in relation to burnout, using a 4-point Likert scale. The scoring system was as follows: 1 for “not relevant,” 2 for “weakly relevant,” 3 for “relevant,” and 4 for “strongly relevant.” The item-level content validity index (I-CVI) and the average scale-level content validity index (S-CVI/Ave) were calculated. A questionnaire was considered to have good content validity if the I-CVI was greater than or equal to 0.78 and the S-CVI/Ave was greater than or equal to 0.90 (20).

##### 2.4.4.2 Structural validity

Exploratory factor analysis was conducted using principal component analysis with maximum variance orthogonal rotation to extract common factors with eigenvalues greater than or equal to 1. An item was retained if its factor loading was >0.4 (21).

#### 2.4.5 Reliability test

##### 2.4.5.1 Internal consistency reliability

A Cronbach's α coefficient of 0.80 or higher is generally considered indicative of a scale's usability (22).

##### 2.4.5.2 Split-half reliability

The Spearman-Brown coefficient was used to assess the split-half reliability of the scale. A split-half reliability >0.80 indicates that the questionnaire has usable value (23).

##### 2.4.5.3 Test-retest reliability

The Pearson correlation coefficient was calculated for the questionnaire scores of 30 operating room nurses assessed on two occasions. A correlation coefficient >0.70 indicates a high level of consistency between pre- and post-measurement, demonstrating good stability of the scale (24).

## 3 Results

### 3.1 Cultural commissioning and pre-survey results

Based on the results of the previous survey, the following adjustments were made: Entry 5 “I get annoyed if there are no standard environmental conditions (such as air conditioning, lighting, temperature) in the operating room” was modified to “I would feel upset if the operating room lacked standard environmental conditions, such as air quality, lighting, temperature, and humidity.” Entry 9 “I get annoyed if the work schedule of the operating room is irregular,” was revised to “I would feel irritated if the working hours in the operating room were irregular.” Entry 19, “When I compare my conditions (economic, social, etc.) with the surgeon, I get disappointed,” was revised to “I felt disappointed when I realized that there was a gap between my condition (economic and social status, etc.) and that of surgeons.” entry 27, “In taking care of patients with emergency conditions, I tolerate more mental and emotional pressure,” was modified to “I face more mental and emotional stress when dealing with patient emergencies or when caring for acutely and critically ill patients.” Additionally, Entry 32, “Te behavior of head nurse in the operating room with surgeons and nurses is discriminatory,” was changed to “The head nurse treated surgeons and nurses differently.”

After cultural adjustments and a pre-survey, only a few entries underwent linguistic revisions, and no content was removed from the questionnaire. The scale maintained a moderate number of entries, with participants taking ~6 to 8 min to complete the questionnaire.

### 3.2 General information of the respondents

The results of the general information survey are presented in Table 1. The majority of participants were female, with age distribution as follows: 36.6% were aged 20–30 years, 52.8% were aged 31–40 years, 9.7% were aged 41–50 years, and 0.9% were over 50 years old. In terms of marital status, 72.6% were married, 25.6% were unmarried, and 1.8% were divorced. Regarding educational attainment, 14.2% held a specialist degree, 84.0% had a master's degree, and 1.8% possessed a postgraduate degree and so on.

### 3.3 Item analysis results

The results of the item analysis indicated that the two groups demonstrated a decision value ranging from 5.92 to 20.55 for each entry, as determined by an independent samples *t*-test (all *P* < 0.001), suggesting that all items on the scale were well differentiated. Pearson correlation analysis revealed that for questionnaire entry one, “I can't accept the decision made by a lay person in the surgical suite,” the correlation coefficient was 0.336, leading to its deletion from the scale. In contrast, the correlation coefficients for the remaining scale entries in relation to the total score ranged from 0.602 to 0.807 (all *P* < 0.001), indicating good differentiation, and these items were retained.

### 3.4 Reliability test results

The Cronbach's alpha coefficient for the Chinese version of the Operating Room Nurses' Burnout Scale was 0.968. The Cronbach's alpha coefficients for each dimension—personal factors, interpersonal factors, occupational nature factors, and organizational factors—were 0.907, 0.897, 0.908, and 0.928, respectively. The test-retest reliability for the total scale was 0.974, with the test-retest reliabilities for the four dimensions being 0.933, 0.905, 0.903, and 0.934. The split-half reliability of the total scale was 0.925. All of these values were statistically significant (*P* < 0.001).

### 3.5 Results of validity analysis

#### 3.5.1 Content validity

The results of this study indicated that the scale demonstrated a content validity index (I-CVI) ranging from 0.849 to 1.000, with a scale-level content validity index (S-CVI/Ave) of 0.965.

#### 3.5.2 Structural validity

The results of the exploratory factor analysis indicated a KMO value of 0.968, suggesting that the sample was appropriate for factor analysis. Additionally, the approximate chi-square value from Bartlett's sphericity test was 11,288 (*P* < 0.01), further confirming the suitability of the data. A total of five common factors with eigenvalues >1 were extracted, accounting for a cumulative variance contribution of 65.9%. However, the fifth factor comprised only entries one and five, with the correlation coefficient for entry one being lower than 0.4. As a result, entry one was deemed suitable for deletion, while entry five was merged into the organizational factor following expert discussion. The dimensions were subsequently renamed as organizational, occupational nature, interpersonal, and personal factors, as shown in Table 2. This scale is in the Appendix 2.

**Table 2:** Factor loading values of each item of the Burnout Scale for Operating Room Nurses (*n* = 445).

**Items**	**Personal factors**	**Interpersonal factors**	**Factors of the nature of the occupation**	**Organi-zational factors**
13	**0.527**			
14	**0.454**			
15	**0.463**			
17	**0.476**			
18	**0.551**			
19	**0.480**			
20	**0.641**			
21	**0.436**			
22		**0.604**		
23		**0.737**		
24		**0.758**		
25		**0.802**		
26		**0.444**		
32		**0.769**		
33		**0.786**		
34		**0.687**		
16			**0.609**	
27			**0.557**	
28			**0.539**	
29			**0.572**	
30			**0.681**	
31			**0.626**	
2				**0.507**
3				**0.522**
4				**0.584**
5				**0.629**
6				**0.690**
7				**0.656**
8				**0.676**
9				**0.676**
10				**0.524**
11				**0.432**
12				**0.448**

Bold: Factor load value of each entry.

## 4 Discussion

### 4.1 The Chinese version of the Operating Room Nurse Burnout Scale has good reliability

Reliability refers to the consistency and stability of measurement results (25). It is generally accepted that a Cronbach's alpha coefficient >0.8 and a test-retest reliability >0.75 indicate good scale reliability, while a Cronbach's alpha coefficient above 0.9 indicates very good scale reliability (22, 23).

In the case of the Chinese version of the burnout scale for operating room nurses, the total scale had a Cronbach's alpha coefficient of 0.968. The coefficients for the individual dimensions ranged from 0.897 to 0.928, and the test-retest reliability was 0.974. Specifically, the Cronbach's alpha coefficients for all dimensions, except for the interpersonal factors, ranged from 0.903 to 0.934. The interpersonal relationship factor had a Cronbach's alpha of 0.897. All other results exceeded 0.9 and were close to the original scale, which had a Cronbach's alpha of 0.937 and a test-retest reliability of 0.946.These results indicate that the scale demonstrates good internal consistency and stability.

### 4.2 The Chinese version of the Operating Room Nurse Burnout Scale has good validity

Validity refers to the accuracy and relevance of questionnaire measurements (26). Typically, content validity and construct validity are used for evaluation. Content validity assesses the correlation between the scale items and the underlying dimensions of the scale. In this study, the item-level Content Validity Index (I-CVI) ranged from 0.849 to 1.000, and the Scale-Level Content Validity Index (S-CVI/Ave) was 0.965, both of which met the required standards, indicating that the content validity of the scale was strong.

Following exploratory factor analysis, five factors were initially extracted. However, the correlation coefficient for item 1, “I can't tolerate the decisions made by non-professionals in the operating room,” was lower than 0.4, leading to its removal based on the cultural context of our country. After consultations with experts, item 5 was integrated into the fourth dimension. As a result, the final structure comprised four dimensions: personal factors, interpersonal factors, occupational factors, and organizational factors. Some items were assigned to dimensions that differed from those in the original scale, likely due to variations in cultural background, medical systems, and social perceptions both domestically and internationally.

The cumulative variance contribution rate of the scale was 65.9%, exceeding the 50% threshold, and the loading values of each item on their respective factors ranged from 0.436 to 0.802, all above 0.4. This suggests that the scale's structure is stable.

### 4.3 Burnout scale for operating room nurses has good application value

Research on burnout in China began relatively recently, primarily focusing on cross-sectional studies, while intervention studies remain limited and have not yet established a robust theoretical foundation (27). Most scholars rely on the three-dimensional theory proposed by Maslach for their research, with the MBI series of scales reflecting the nature and manifestations of burnout. The Operating Room Nurse Burnout Scale takes the factors affecting burnout as its entry point, enabling researchers to better explore the causes of burnout, identify specific manifestations, and understand the underlying mechanisms. This adaptation of the scale is significant for advancing the theory of burnout in China.

Burnout is a common occupational hazard and a significant challenge faced by nursing staff, severely impacting their physical and mental health (28). It should be viewed as a characteristic of the workgroup rather than merely an individual syndrome, reflecting a breakdown in the relationship between the individual and their work (29). The formation of burnout is complex, influenced by various factors. In the unique context of the operating room, the closed working environment, intricate interpersonal dynamics, and the specific nature of the job contribute to burnout in operating room nurses, potentially differing from the factors affecting nurses in general wards. The burnout experienced by operating room nurses is shaped by interpersonal, occupational, organizational, and personal factors (30). Studying these influences can provide a foundation for developing appropriate interventions.

Teymoori et al. (15) explored the concept and influencing factors of burnout from the perspective of operating room nurses, leading to the creation of the Burnout Scale for Operating Room Nurses. The content of this scale is closely aligned with the work responsibilities of operating room nurses and directly reflects the factors contributing to their burnout. Consequently, the scale serves not only to assess the degree of burnout among operating room nurses but also to inform the development of targeted interventions.

Surgery is fundamentally a team process, and operating room nurses are vital members of the healthcare team, playing crucial roles in maintaining patient safety, ensuring the smooth progress of surgery, facilitating communication and coordination, and controlling surgical infections. The prevalence of burnout among operating room nurses is concerning, as it can lead to diminished enthusiasm for their work, reduced efficiency, and impaired ability to manage job demands, ultimately affecting surgical department operations and patient safety (31). Therefore, it is essential to prioritize the physical and mental wellbeing of operating room nurses to mitigate burnout.

However, there is a scarcity of domestic studies on burnout among operating room nurses, likely due to the absence of specific research tools. Thus, developing or introducing a dedicated assessment tool for burnout in this population is critical for advancing research in this area. The scale is user-friendly and can effectively evaluate burnout levels among operating room nurses, providing a basis for designing interventions based on its dimensions. In this study, we adhered strictly to the scale introduction process, leading to the Chinese adaptation and subsequent testing for reliability and validity. The results indicated that the Chinese version of the Operating Room Nurse Burnout Scale comprises four dimensions with 33 items, demonstrating good reliability and validity.

### 4.4 Limitations and future research

The sample for this study was exclusively drawn from tertiary hospitals in Anhui Province, representing a geographical limitation. Future research is recommended to expand the survey scope to include nurses from various regions. This approach would further validate the reliability of the Chinese version of the Operating Room Nurses Burnout Scale and promote its broader application within the nursing field.

## Data Availability

The original contributions presented in the study are included in the article/Supplementary material, further inquiries can be directed to the corresponding author.
