# Supplementary material for: Sinicization and validation of occupational burnout scale for nurses in operating room
Source: Front Public Health. 2025 Mar 19;13:1559204. doi: 10.3389/fpubh.2025.1559204 (PMC11961642; doi:10.3389/fpubh.2025.1559204)
Supplement: Supplementary file 1 [file Table_1.docx]

The Questionnaire Of Burnout Factors In Operating Room Nurses（Primary scale）

| Item Number | **The title of the item** |
| --- | --- |
| Factor 1: organizational factors |  |
| 1 | When the decision in the operating room is made by non-specialists, it is unbearable for me |
| 2 | I am under pressure because the organization does not support me enough in the work environment |
| 3 | Lack of job promotion makes me feel unmotivated |
| 4 | If there are no proper instruments and equipment in the operating room, I bear more work pressure |
| 5 | I get annoyed if there are no standard environmental conditions (such as air conditioning, lighting, temperature) in the operating room |
| 6 | Te disproportion between salary and work duties makes me demotivated |
| 7 | Unrelated tasks in the operating room make me tired |
| 8 | Due to the insufcient number of nurses, I bear more pressure |
| 9 | I get annoyed if the work schedule of the operating room is irregular |
| 10 | In case of insufcient skill of the surgeon, I tolerate more pressure |
| 11 | If there is no empathy between operating room nurses, I get annoyed |
| 12 | If my colleagues are insufciently skilled in their duties, I get more tired |
| Factor 2: individual factors |  |
| 13 | It is hard for me to bear working in the closed space of the operating room and not being able to move between departments |
| 14 | I get upset when I see people’s misconceptions about my job |
| 15 | I feel inefcacity because I don’t imagine a better work future for myself |
| 16 | When a patient dies in the operating room, I endure severe mental and emotional pressure |
| 17 | I get tired when I do repetitive activities in the operating room |
| 18 | I sufer from not being able to show my true feelings in the operating room |
| 19 | When I compare my conditions (economic, social, etc.) with the surgeon, I get disappointed |
| 20 | I get annoyed when have to keep silent when I see the inappropriate behavior of managers |
| 21 | Due to economic problems, I have to work more |
| Factor 3: interpersonal factors |  |
| 22 | If I don’t pay my salary on time, I will be pressured |
| 23 | I bear a lot of pressure in the operating room if the surgeon become angry |
| 24 | When the self-esteem and personality of the nurses are ignored by the surgeon, I get annoyed |
| 25 | Due to the bullying and dominance of the surgeon in the operating room, I bear more work pressure |
| 26 | Te presence of hypocrisy behavior in colleagues is painful for me |
| Factor 4: occupational nature factors |  |
| 27 | In taking care of patients with emergency conditions, I tolerate more mental and emotional pressure |
| 28 | Te possibility of physical and mental problems due to the stress in the operating room worries me |
| 29 | Unexpected surgical events (such as lost sponges or surgical instruments) cause more work pressure on me |
| 30 | I bear a lot of pressure while working at night shif |
| 31 | It is difcult for me to move heavy instruments and equipment |
| Factor 5: managerial factors |  |
| 32 | Te behavior of head nurse in the operating room with surgeons and nurses is discriminatory |
| 33 | Lack of attention to my physical and mental conditions by head nurse is painful for me |
| 34 | If I criticize of the head nurse, more pressure will be imposed on me |
