# Supplementary material for: Sinicization and validation of occupational burnout scale for nurses in operating room
Source: Front Public Health. 2025 Mar 19;13:1559204. doi: 10.3389/fpubh.2025.1559204 (PMC11961642; doi:10.3389/fpubh.2025.1559204)
Supplement: Supplementary file 2 [file Table_2.docx]

手术室护士职业倦怠量表（中文版）

| **条目** | **具体内容** |
| --- | --- |
| 1、个人因素 |  |
| 1 | 手术室这种的封闭工作环境，且不能随意出入其他手术间，这对于我来说是难以忍受的 |
| 2 | 当我发现别人对我的工作有误解时，我就会感到很难过 |
| 3 | 如果我对于自己的工作发展前景憧憬不好，我将十分沮丧 |
| 4 | 手术室里重复性的工作使我感到很疲倦 |
| 5 | 我因不能在手术室里表达自己的真实感受而痛苦 |
| 6 | 当我发现我的情况（经济社会地位等）与外科医生有差距时，我感觉很失望 |
| 7 | 当我看见领导不合时宜的表现而我不得不保持沉默时，我将很恼火 |
| 8 | 由于某些经济问题，我不得不多工作 |
| 2、人际关系因素 |  |
| 9 | 如果我的工资没有按时发放，我就会有压力 |
| 10 | 如果外科医生在手术间发脾气，我将承受很大的压力 |
| 11 | 当护士的自尊和人格被外科医生忽视时，我将十分恼火。 |
| 12 | 如果外科医生在手术间里恃强凌弱，我就会承受更多的工作压力。 |
| 13 | 同事间的虚伪行为让我很痛苦 |
| 14 | 护士长区别对待外科医生和护士 |
| 15 | 护士长不关心我的身心状况让我很难过 |
| 16 | 如果我指责护士长，我将会面临更大的压力 |
| 职业性质因素 |  |
| 17 | 当病人在手术室死亡，我将承受严重的生理和心理压力 |
| 18 | 在处理患者的紧急情况时或面对急危重症患者，我面临着更大的心理压力和情感压力 |
| 19 | 我很担心由工作带来的压力会导致我出现各种身体和心理问题 |
| 20 | 一些突发意外事件（例如纱布丢失或者手术器械丢失）将让我产生更多的压力 |
| 21 | 在上夜班时我承受的压力更大 |
| 22 | 搬运重型仪器和设备对我来说是很困难的 |
| 组织因素 |  |
| 23 | 由于科室在工作环境方面没有给予我足够的支持，这使我感到有压力 |
| 24 | 缺乏晋升机会让我感到没有动力 |
| 25 | 如果手术室没有合适的仪器设备，我承受的工作压力更大 |
| 26 | 如果手术室没有标准的环境条件（如空气质量、照明、温湿度），我会很生气 |
| 27 | 工资和工作职责之间的不平衡让我失去了动力 |
| 28 | 手术室里承担工作职责以外的任务让我很疲劳 |
| 29 | 护士人力资源不足导致我的压力更大 |
| 30 | 如果手术室的工作时间不规律，我会很恼火 |
| 31 | 如果外科医生的技术不够熟练，我将承受更多的压力 |
| 32 | 如果手术室护士之间没有同理心，我将会十分烦恼 |
| 33 | 如果我的同事缺乏足够的专业知识和临床技能，我会感到更疲惫 |

Burnout Scale for Operating Room Nurses (Chinese - English translation)

| **条目** | **具体内容** |
| --- | --- |
| 1、**personal factors** |  |
| 1 | The closed working environment of the operating room and the lack of access to other operating rooms is intolerable to me. |
| 2 | I feel disheartened when I realize that people misunderstand my work. |
| 3 | I would feel quite frustrated if I had a negative outlook on my job development prospects. |
| 4 | I am feeling weary of the repetitive tasks in the operating room. |
| 5 | I struggle with not being able to express my true feelings in the operating room. |
| 6 | I feel disappointed when I recognize the gap between my situation—such as my economic and social status—and that of the surgeon. |
| 7 | I feel annoyed when I witness leaders behaving inappropriately and have to remain silent. |
| 8 | I have to work harder due to some financial challenges. |
| 2、**Interpersonal factors** |  |
| 9 | I will feel stressed if my salary is not paid on time. |
| 10 | I will feel stressed if the surgeon loses his temper in the operating room. |
| 11 | I will feel very annoyed when a surgeon overlooks a nurse's self-esteem and personality. |
| 12 | If surgeons bully others in the operating room, I'll feel increased pressure to perform my job. |
| 13 | Hypocritical behavior among coworkers is upsetting to me. |
| 14 | The head nurse treats surgeons and nurses differently. |
| 15 | The head nurse's lack of concern for my physical and mental well-being makes me feel sad. |
| 16 | I will feel more pressure if I accuse the head nurse. |
| **Factors of the nature of the occupation** |  |
| 17 | When a patient dies in the operating room, I experience significant physical and psychological stress. |
| 18 | I experience greater psychological and emotional stress when dealing with patient emergencies or critically ill patients. |
| 19 | I am worried that the stress from my work will lead to various physical and psychological issues. |
| 20 | Unforeseen events, such as the loss of gauze or surgical instruments, increase my stress levels. |
| 21 | I experience increased stress during night shifts. |
| 22 | I find it difficult to lift heavy instruments and equipment. |
| **Organizational factors** |  |
| 23 | The lack of adequate support from the department regarding the working environment contributes to my stress. |
| 24 | The lack of promotion opportunities makes me feel unmotivated. |
| 25 | If there are no suitable instruments and equipment in the operating room, I feel increased work pressure. |
| 26 | I would feel upset if the operating room lacked standard environmental conditions, such as air quality, lighting, Temperature and humidity, and humidity. |
| 27 | The disparity between pay and job duties de-motivates me. |
| 28 | Taking on tasks outside of my job responsibilities in the operating room makes me feel fatigued. |
| 29 | The lack of nursing staff increases my stress levels |
| 30 | I would feel irritated if the working hours in the operating room were irregular. |
| 31 | I would feel more stressed if the surgeons were not sufficiently skilled. |
| 32 | I would be very annoyed if there were no empathy among nurses in the operating room. |
| 33 | I would feel more exhausted if my colleagues lacked adequate professional knowledge and clinical skills. |
